# The analysis of heterotaxy patients reveals new loss-of-function variants of *GRK5*

**DOI:** 10.1038/srep33231

**Published:** 2016-09-13

**Authors:** Davor Lessel, Tariq Muhammad, Teresa Casar Tena, Barbara Moepps, Martin D. Burkhalter, Marc-Phillip Hitz, Okan Toka, Axel Rentzsch, Stephan Schubert, Adelheid Schalinski, Ulrike M. M. Bauer, Christian Kubisch, Stephanie M. Ware, Melanie Philipp

**Affiliations:** 1 Institute of Human Genetics, University Medical Center Hamburg-Eppendorf, 20246 Hamburg, Germany; 2 Department of Pediatrics, Indiana University School of Medicine, Indianapolis, IN 46202, USA; 3 Institute for Biochemistry and Molecular Biology, Ulm University, 89081 Ulm, Germany; 4 Institute of Pharmacology and Toxicology, Ulm University Medical Center, 89081 Ulm, Germany; 5 Department of Congenital Heart Disease and Pediatric Cardiology, University Hospital Schleswig-Holstein, 24105 Kiel, Germany; 6 Friedrich-Alexander-Universität Erlangen-Nürnberg (FAU), Department of Pediatric Cardiology, 91054 Erlangen, Germany; 7 Department of Pediatric Cardiology, Saarland University Medical Center, 66421 Homburg, Germany; 8 German Heart Institute Berlin, Department of Congenital Heart Disease and Pediatric Cardiology, 13353 Berlin, Germany; 9 Competence Network for Congenital Heart Defects/National Register for Congenital Heart Defects, 13353 Berlin, Germany

## Abstract

G protein-coupled receptor kinase 5 (GRK5) is a regulator of cardiac performance and a potential therapeutic target in heart failure in the adult. Additionally, we have previously classified GRK5 as a determinant of left-right asymmetry and proper heart development using zebrafish. We thus aimed to identify GRK5 variants of functional significance by analysing 187 individuals with laterality defects (heterotaxy) that were associated with a congenital heart defect (CHD). Using Sanger sequencing we identified two moderately frequent variants in GRK5 with minor allele frequencies <10%, and seven very rare polymorphisms with minor allele frequencies <1%, two of which are novel variants. Given their evolutionarily conserved position in zebrafish, in-depth functional characterisation of four variants (p.Q41L, p.G298S, p.R304C and p.T425M) was performed. We tested the effects of these variants on normal subcellular localisation and the ability to desensitise receptor signalling as well as their ability to correct the left-right asymmetry defect upon Grk5l knockdown in zebrafish. While p.Q41L, p.R304C and p.T425M responded normally in the first two aspects, neither p.Q41L nor p.R304C were capable of rescuing the lateralisation phenotype. The fourth variant, p.G298S was identified as a complete loss-of-function variant in all assays and provides insight into the functions of GRK5.

Heterotaxy describes a very rare condition with an incidence of 1:10,000^1^. It is characterized by a deviation from the normal situs (situs solitus), a term which describes the normal asymmetric distribution of internal organs such as the heart, liver, spleen or pancreas. In contrast to individuals with situs inversus, which is a complete mirror image of organ placement, patients with heterotaxy appear to have a random distribution of their internal organs with for instance their heart apex being oriented rightward (dextrocardia), absence of the spleen (asplenia) or inverted gut looping (malrotation)^2^. As a consequence several complications can arise with congenital heart defects (CHD) being the most common and severe. Such CHDs with concomitant situs anomalies are clinically and genetically heterogeneous disorders. Oftentimes they consist of complex cardiovascular malformations such as transposition of the great arteries or double outlet right ventricle^3^.

G protein-coupled receptor kinase 5 (GRK5) is a versatile regulator of cellular signalling events, which also functions as a critical modulator of cardiac performance in the adult^4^. It further represents the only cardiac GRK for which clinically relevant genetic variations have been identified. The best-characterised variant so far is a single nucleotide polymorphism (SNP) rs17098707 resulting in an amino acid exchange in the N terminus. This p.Q41L variant has been found beneficial in a certain population of heart failure patients^5^ and was associated with fewer adverse cardiovascular events in hypertensive patients^6^. However, its full impact on signalling has not yet been clarified. Moreover, it has also been shown that p.Q41L may predispose to certain cardiac conditions such as Takotsubo cardiomyopathy^7^,^8^. Similarly, intronic SNPs which have been associated with atrial fibrillation after coronary bypass surgery^9^ will require further genetic and biochemical analyses to uncover their apparently clinically relevant but mechanistically unknown function.

In addition to its well-accepted function in the adult heart, we have recently shown that GRKs of the GRK4-6 family play a role during embryonic development^10^,^11^. Mice lacking both, GRK5 and GRK6 are embryonically lethal. Furthermore, depletion of either mouse GRK5 or its closest zebrafish homolog termed Grk5l results in elevated mTOR activity, which in turn impairs leftward gene expression in the lateral plate mesoderm, where the heart progenitors reside. As a consequence, a highly conserved pathway required for left-right patterning across species cannot be established properly, and abnormal internal asymmetry and cardiac development can be observed in zebrafish^10^.

These findings prompted us to screen *GRK5* in DNA samples collected from 69 patients of German origin and in DNA samples obtained from 118 patients in the United States diagnosed with heterotaxy and concomitant CHD, in whom we previously excluded mutations in known heterotaxy genes. We detected functionally significant single nucleotide variants which suggest a potential contribution to the development of heterotaxy.

## Results

### Identification of *GRK5* Sequence Variants

**Figure 1:**
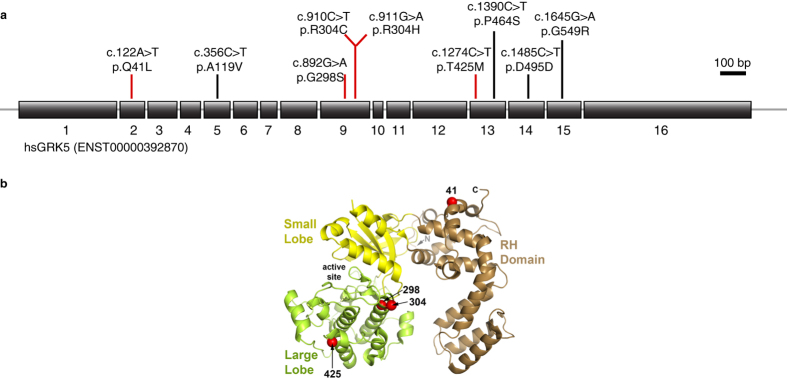
Variants of human GRK5 identified in heterotaxy patients. (**a**) Cartoon displays the 16 exons and the detected genetic variations as well as their respective location within the coding region. The variants are shown at the nucleotide levels (e.g. c.122A < T) as well as the subsequent amino acid change in the protein (e.g. p.Q41L). Red bars mark variants more closely characterised in this study. The p.T425M variant of GRK5 identified in human patients corresponds to the p.S425M variant of zebrafish Grk5l used in this work. (**b**) Numbers and red spheres indicate location of relevant variants in the structure of bovine GRK5 (PDB entry 4WNK). Only position 298 is absolutely conserved as glycine in GRKs. Image provided by John J. G. Tesmer, University of Michigan, Ann Arbor.

Sequencing of 69 individuals with CHD (Supplementary Table S1) and concomitant situs anomalies from the initial cohort identified 7 rare heterozygous variants (Table 1 and Supplementary Table S2). Their position in the gene and protein is shown in Fig. 1 and their minor allele frequencies are given in Table 2. Two variants, namely p.Q41L (rs17098707) and p.R304H (rs2230349), are moderately frequent with a minor allele frequency of <10%. Three additional variants, which are p.A119V (rs55980792), p.G298S (rs140946236) and p.D495D (rs149159651), are rare polymorphisms with minor allele frequencies of <1% (Table 2). In addition, we observed two novel coding variants, according to both ExAC Browser and dbSNP build 139, namely p.P464S and p.G549R. Notably, we detected two rare heterozygous variants in two affected individuals (Table 1). In individual 43 we detected both p.A119V and p.G298S, though further analysis revealed both to be paternally inherited. Individual 38 bears both p.R304H and p.G549R, however DNA samples from parents were not available for testing.

These data prompted us to analyse those exons of *GRK5* in which we detected rare variants in an independent cohort of 118 affected individuals. In addition to identifying individuals bearing p.Q41L and p.R304H, we found two further variants, p.R304C (rs145397190) and p.T425M (rs77323445) with minor allele frequencies of <1% (Table 2). In summary, we found two moderately frequent and seven rare variants in our cohorts of CHD patients with concomitant situs anomalies. All patients except for one carrying p.Q41L were heterozygous carriers of the *GRK5* variants. To determine the possible effect of these single nucleotide variants on GRK function, analysis was performed in heterologous cell systems and zebrafish. We concentrated our analysis on four amino acid changes. Substitutions p.G298S and p.R304C were chosen because both mutated residues were conserved in zebrafish (see protein alignment in Supplementary Figure S1). In addition, we included p.T425M. In zebrafish the threonine at position 425 is a serine, which is also an uncharged amino acid that can be phosphorylated by serine-threonine kinases. Hereafter, this variant will be named p.S425M. These three variants were further chosen as they were predicted to be possibly damaging for GRK5’s function (Table 2). For such predictions we applied SIFT and Polyphen-2 (Polymorphism Phenotyping v2) algorithms, which calculate how harmful a genetic variant is likely to be based on the structure, function and degree of amino acid conservation^12^,^13^. The lower a SIFT score, the higher the likelihood that a variant is detrimental. We also included the previously reported variant p.Q41L which had been proposed to be beneficial to cardiac function under some conditions, but deleterious to the heart under other^5^,^6^,^7^,^8^. We refrained from analysing the two novel variants as the closest zebrafish homolog of human GRK5 already contains a serine corresponding to position 464 in humans and arginine at position relative to position 549 in humans (Supplemental Figure S1). The position of the detected variants with respect to the exon sequence of GRK5 is depicted in Fig. 1, which also highlights the position of the four variants analysed in more detail in the crystal structure of GRK5.

### The Impact of GRK5 Variants on GPCR Desensitisation

**Figure 2:**
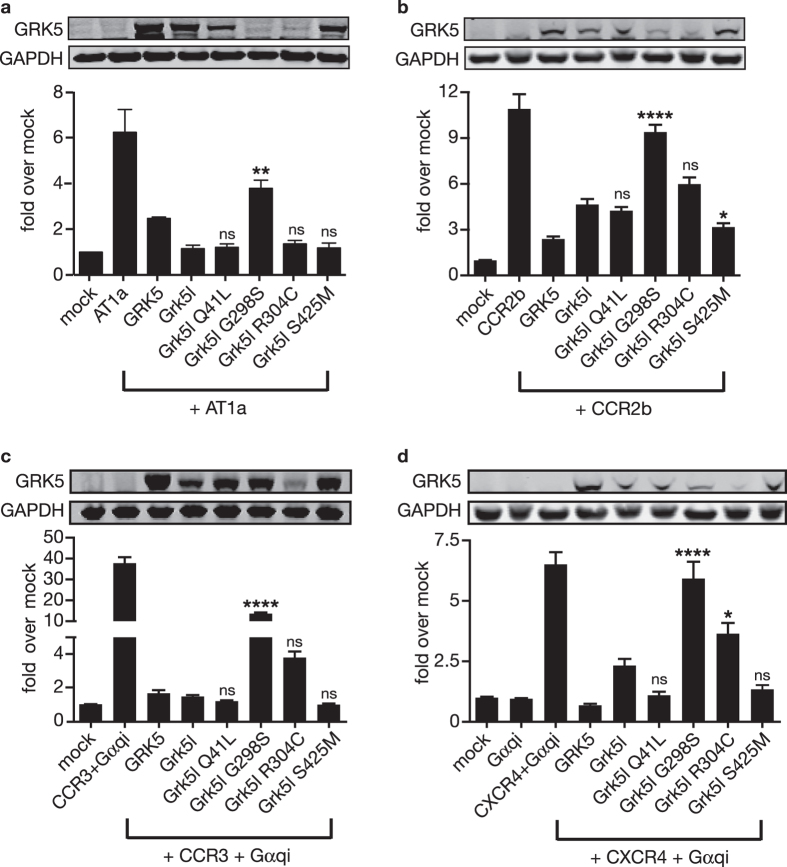
Mutations in Grk5l alter its ability for receptor desensitisation. We tested the ability of Grk5l variants to terminate signalling of four different receptors in a luciferase reporter assay that is based on the Gq-Rho-SRE signal transduction axis. Individual receptors were transiently expressed in HEK293 cells together with wild-type Grk5l or different variants thereof. As positive control for receptor desensitisation, bovine GRK5 was co-expressed with the receptor. All panels show representative blots. One-way ANOVA was applied and all variants were compared to wild-type Grk5l; *p < 0.05; **p < 0.01; ***p < 0.001; ****p < 0.0001; ns, not significant. Bar graphs display mean ± SEM of three independent experiments done in triplicates. (**a**) Murine AT1a receptor stimulated with 30 μM angiotensin II: All variants except for p.G298S display the same efficiency in the desensitisation of AT1a receptors. (**b**) Human CCR2b receptor stimulated with 50 nM CCL2: The p.G289S shows significantly reduced ability to terminate signalling compared to wild-type Grk5l, while p.S425M is even more potent. (**c**) Human CCR3 receptor stimulated with 50 nM CCL11: As CCR3 is not G_q_-coupled a Gα_qi5_ construct was co-transfected, which enables G_i_-receptors to stimulate G_q_ signalling cascades. Grk5l inhibits signalling to a similar extent as bovine GRK5. The p.G298S variant lacks the ability to desensitise CCR3 receptors, while the lower inhibition seen by p.R304C may be accounted to lower expression levels. (**d**) Human CXCR4 stimulated with 50 nM CXCL12: As CXCR4 is not G_q_-coupled a Gα_qi5_ construct was co-transfected, which enables G_i_-receptors to stimulate G_q_ signalling cascades. On its own, Gα_qi5_ does not alter receptor signalling. p.G298S and also p.R304C are less effective in inhibiting receptor signalling, whereas p.Q41L and p.S425M appeared to be more potent than wild-type Grk5l.

In the classical paradigm of G protein-coupled receptor (GPCR) signalling, agonist binding induces a conformational change in receptors that in turn activates signalling of the heterotrimeric G protein^14^. GRKs serve as the terminating enzymes of active signalling. To address this, we examined their effect on signalling of murine angiotensin II receptor type1a (AT1a) (Fig. 2a) and the human chemokine receptors CCR2b (Fig. 2b), CCR3 (Fig. 2c) and CXCR4 (Fig. 2d). Both, AT1a and CXCR4 receptors have previously been shown to be regulated by GRKs, including GRK5^15^,^16^. For all four receptors we made use of a luciferase-based system, in which the activation of the transcriptional regulatory element SRE.L by a Gα_q_ coupled receptor is measured and which we had established for the measurement of G protein-mediated chemokine receptor signalling^17^,^18^. Since CCR3 and CXCR4 predominantly couple to Gα_i_, measurement of signalling was facilitated by co-expression with a chimeric Gα_qi5_, in which the last five amino acids of Gα_q_ are exchanged for those of Gα_i_. By this change, signalling of Gα_i_ coupled receptors can be redirected to Gα_q_ proteins^19^,^20^. In this setting, stimulation of either receptor activates Rho GTPases, which cause cytoskeletal rearrangements and transcriptional activity of SRF, for which SRE.L functions as readout^21^,^22^,^23^.

For all tested receptors agonist stimulation robustly induced activation of luciferase indicating that active signalling is conferred. Co-expression of bovine GRK5 abrogated signalling, which could be similarly observed for co-expression of Grk5l. p.Q41L which has been shown to be more potent in desensitising β_2_ adrenergic receptors^24^, desensitised the receptors to a similar if not almost greater extent than wild-type Grk5l. p.G298S, on the other hand, failed to terminate G protein signalling under all experimental conditions. p.R304C as well as p.T425M both retained the ability for receptor desensitisation although to different extends. Most likely the reduced capability of p.R304C to block CCR3 (Fig. 2c) and CXCR4 (Fig. 2d) signalling was due to the lower expression levels, which could not be elevated by increasing the amount of transfected plasmid or by different plasmid preparations. We also tested the effect of Gα_qi5_ alone, which produced only marginal, if any activation of SRE.L. Taken together, we confirmed that p.Q41L is as effective as wild-type GRK in receptor desensitisation and found that the glycine at position 298 is crucial for receptor desensitisation, indicating a functional effect of this variant in GPCR signalling.

### Subcellular Localisation of GRK5 Depends on Glycine 298

**Figure 3:**
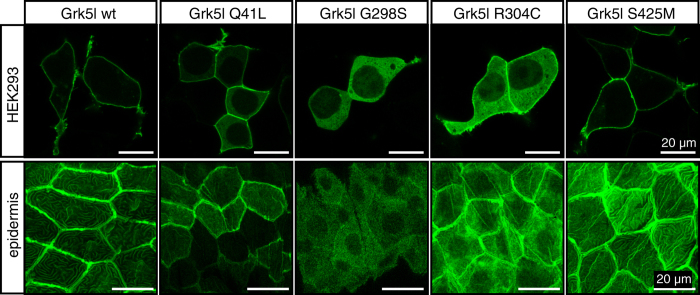
Subcellular distribution of Grk5l variants. Upper panel: HEK293 cells were transiently transfected with GFP fusion constructs of wild-type or mutated Grk5l (cloned into pEGFP-N3) and analysed using confocal microscopy. Representative images of three transfections shown. Lower panel: Expression of Grk5l variants in zebrafish produced subcellular distribution of the kinases similar to the results in HEK293 cells. Images show expression in the epidermis. At least five different embryos each from two different injection days were assessed.

One characteristic of GRK5 is the predominant association with the cytosolic side of the plasma membrane through N- and C-terminally located basic or hydrophobic amino acids^25^,^26^,^27^. Considering that this membrane localisation is a crucial requirement for receptor desensitisation by a GRK we assessed the subcellular localisation of the newly identified GRK5 variants in a heterologous cell system and in zebrafish. Transient transfection of wild-type Grk5l revealed the expected membrane association with little or no expression in the cytosol. Similarly, p.Q41L strongly localised to the plasma membrane with some protein remaining in the cytoplasm. Mutation of glycine 298 to serine, however, completely abrogated membrane association of Grk5l and resulted in cytosolic retention with a small fraction locating to the nucleus. When arginine 304 was exchanged for cysteine the kinase appeared to be evenly distributed between the cytosol and the plasma membrane. In addition, weak fluorescence could be seen in the nucleus. Finally, Grk5l S425M, which had been indistinguishable to wild-type Grk5l in the desensitisation assay, nicely associated with the plasma membrane (Fig. 3 upper panels).

We next wondered whether the same subcellular pattern occurred *in vivo* and injected capped RNA encoding either wild-type Grk5l or one of the four variants fused to GFP into fertilised zebrafish eggs. As readout we analysed GFP fluorescence in the epidermis at 24 hours post fertilisation (hpf). Similar to expression in a heterologous cell system, Grk5l wild-type, p.Q41L and p.S425M localised to the membrane in zebrafish embryos. Expression of p.G298S yielded only cytosolic localisation, while p.R304C was again evenly distributed between cytosol and membrane (Fig. 3 lower panels). These results confirm and extend our desensitisation studies. Moreover, they indicate that membrane association may depend on additional domains within GRK5 in addition to the amphipathic helix.

### Grk5l variants localise to cilia

**Figure 4:**
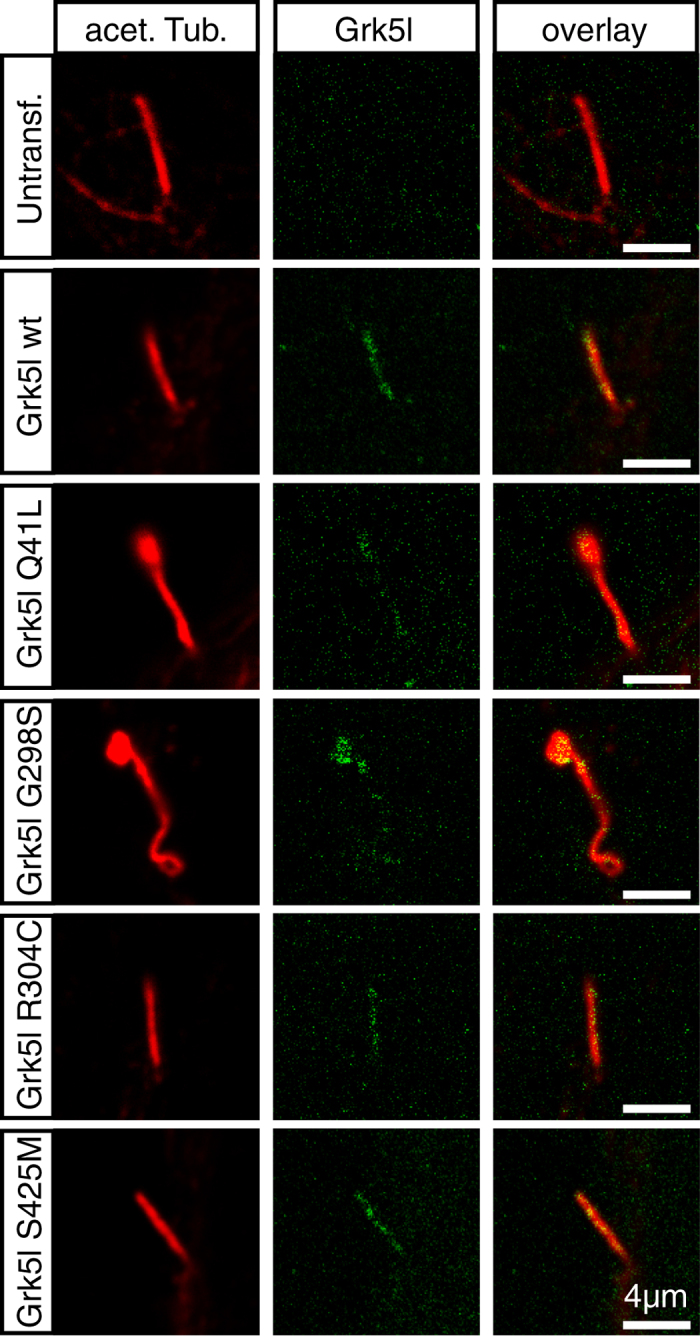
Variants of Grk5l localise to cilia. Human forearm fibroblast cells immortalised by hTERT were transfected with pEGFP-N3 plasmids carrying HA-tagged wild-type or mutated Grk5l cDNA, followed by serum starvation. Cells were then probed for acetylated-tubulin and the HA tag, respectively, to detect primary cilia and expressed Grk5l variants. Assessment by confocal microscopy revealed ciliary localisation of all variants. Acetylated-tubulin is shown in red in panels to the left. Localisation of Grk5l variants are represented in green in middle panels, while panels to the right show an overlay.

Left-right asymmetry and subsequent heart development depend on cilia^2^. Previously, we have found that Grk5l regulates cilia function and that it localizes to cilia^10^. We have thus investigated whether mutation of Grk5l changes ciliary localization. Interestingly and in contrast to the differences in membrane localization, we could detect all four variants in cilia of cultured fibroblasts (Fig. 4) suggesting that any contribution to the development of heterotaxy-driven CHD is likely caused by an inability to regulate signal transduction.

### Impact of GRK5 Variants on Left-Right Asymmetry Development

**Figure 5:**
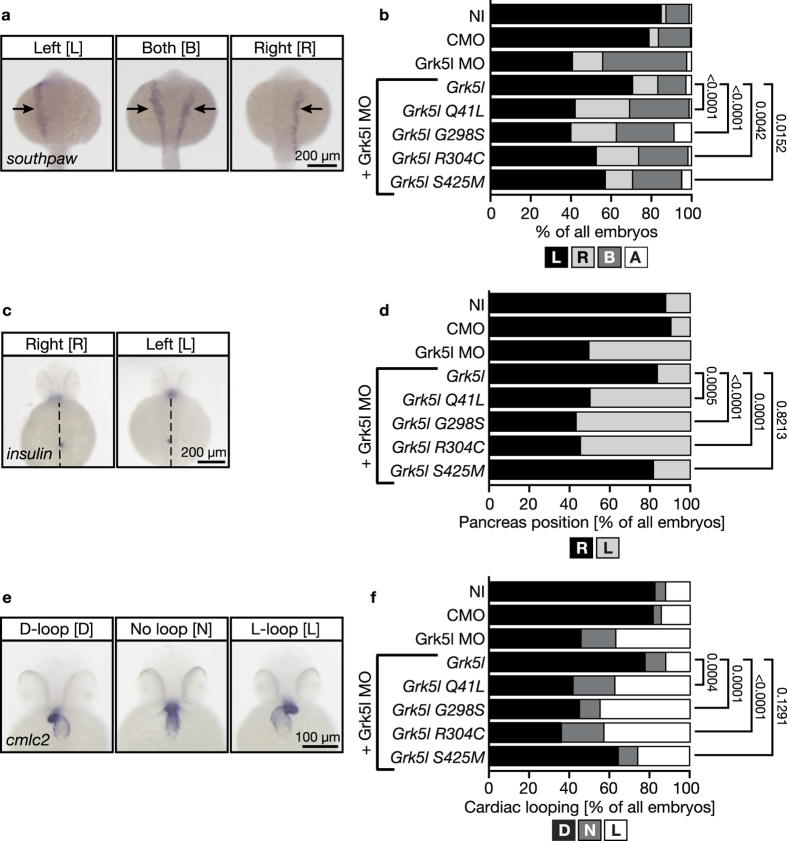
Functional analysis of GRK5 variations in zebrafish. (**a**) Whole mount *in situ* hybridisation of 20–22 somite stage zebrafish embryos for the leftward gene *southpaw* (*spaw*). Embryos were either left uninjected (NI) or were injected with a 5 basepair mismatch control MO (CMO) or a translation blocking MO against Grk5l, respectively. To analyse the functionality of the different Grk5l variants, fertilised eggs were simultaneously injected with capped RNAs for wild-type Grk5l or one of the four variants. Control embryos predominantly express *spaw* in the left lateral plate mesoderm (left panel), while. Grk5l depleted embryos show aberrant *spaw* expression bilaterally or right of the midline (middle and right panel). (**b**) Summary of 4 to 17 independent experiments with 114–432 embryos: embryos were scored for *spaw* expression left (L) or right (R) from the midline, for bilateral (B) or absent expression (A) See supplement for injections of RNA only. (**c**) WMISH of 48 hours post fertilisation (hpf) zebrafish embryos for the *insulin* gene to detect placement of the pancreas upon rescue attempts with Grk5l variant RNAs. In controls, pancreas placement is predominantly on the right side of the midline (indicated by dashed line), while Grk5l depletion renders embryos with left-sided pancreas. (**d**) Summary of 3 to 6 independent experiments with 42–133 embryos: pancreas position is indicated by L (left) or R (right). See supplement for injections of RNA only. (**e**) WMISH of 48 hpf zebrafish embryos for *cardiac myosin light chain 2* (*cmlc2*) to monitor heart looping. Left panel shows a correctly looped heart (D), the middle displays an unlooped heart (N) and the right panel shows an inversely looped heart (L). (**d**) Summary of 3 to 6 independent experiments with 42–133 embryos scored for heart looping after co-injection of Grk5l MO and different rescue RNAs. See supplement for injections of RNA only. In all panels anterior to the top. Fisher’s exact test was applied to compare efficiency of rescue of variant to wt Grk5l and p values are indicated to the right.

Finally, we tested whether any of the GRK5 variants had an impact on the development of left-right asymmetry. Bilateral asymmetry determines the oriented morphogenesis of internal organs with respect to each other, but also within the organs itself, particularly the heart^2^,^3^. Thus, the development of a CHD may be propagated if one of the variants would induce aberrant left-right asymmetry development. First, we monitored the side-specific expression of the lateralisation gene *southpaw* (*spaw*). *Spaw* is expressed exclusively in the left lateral plate mesoderm during somitogenesis^28^. When symmetry breaking fails such as in the case of MO-mediated Grk5l depletion^10^, *spaw* can be found in a bilateral expression or may be solely present on the right site of the body (Fig. 5a). We have shown in our initial study that the asymmetry defect in Grk5l knockdown embryos can be rescued by reconstitution with Grk5l^10^. We have thus tested whether the four variants retained this ability to recue asymmetry. Compared to rescue with wild-type Grk5l, none of the variants could significantly rescue correct *spaw* expression in the lateral plate mesoderm. Nevertheless, p.S425M showed a tendency towards a partial rescue (Fig. 5b). To further investigate whether any of the variants affected situs development, we scored abdominal organ placement by examining the localization of the endocrine pancreas (Fig. 5c,d). Again, co-injection of RNA encoding p.Q41L, p.G298S and p.R304C did not correct pancreas positioning with respect to the midline (Fig. 5d). p.S425M, interestingly and concordant with the tendency of *spaw* rescue in the left lateral plate mesoderm, rescued pancreas placement (Fig. 5d).

The most interesting parameter in the light of this study is the analysis of cardiac looping, which becomes randomized in zebrafish with impaired left-right asymmetry establishment. Under healthy control conditions the zebrafish heart undergoes looping from 36 hours post fertilization (hpf) on. At 48 hpf, the heart is shaped like an S, where the ventricle lies left and above of the atrium (Fig. 5e). When asymmetry is disturbed, inversely looped hearts or hearts, which completely fail to loop can be observed (Fig. 5e). MO-mediated knockdown of Grk5l results in randomization of heart looping with roughly half of the embryos still developing normally looped hearts. Complementation with wild-type Grk5l or p.S425M through co-injection of capped RNA reproducibly corrected heart looping. Co-injection of any of the other variants, however did not rescue indicating that the detected variants may contribute to the development of heterotaxy-driven CHD (Fig. 5f).

Last, but not least, to rule out a potential dominant negative effect of p.Q41L, p.G298S and p.R304C and since the detected patient variants were mostly heterozygous, we injected the respective RNAs into wild-type embryos without Grk5l MO. Embryos injected with either of the three RNAs were indistinguishable from control injected embryos for *spaw* localisation, pancreas placement or heart looping indicating that the rescue experiment results are not explained by variant inhibition of endogenous Grk5l. Interestingly, injection with RNA encoding p.S425M interestingly affected *spaw* expression, although it did not impair asymmetry developmentof developing organs at later stages (Supplementary Figures S2–4). Further studies would be required to better understand the unexpected effect on *spaw*.

## Discussion

The term CHD describes the developmental malformation of the heart and its connected structures. It affects a large number of newborns and differs greatly regarding the type and complexity of malformation and thus severity^29^. Genetically, single gene causes of CHDs such as *TBX5* mutations in Holt-Oram-Syndrome or mutations in the cardiac differentiation factor NKX2.5^30^,^31^ explain a relatively small proportion of all known cases. While the ability to provide a cytogenetic or molecular diagnosis for causes of syndromic or isolated CHD are increasing, up to 80% of cases are still without an etiologic explanation^32^. Furthermore, mutations in single genes cannot explain the variability in the severity of different CHDs. Therefore it is the generally assumed that the majority of CHDs are multifactorial, involving combinatorial genetic and environmental interactions. Heterotaxy spectrum CHD has the highest relative risk amongst CHD subtypes, indicating a strong genetic basis^33^,^34^,^35^. Those defects arise when internal bilateral asymmetry cannot be established. In our previous study, we have established GRK5 as a novel determinant of left-right asymmetry and heart development in mice and zebrafish^10^. Herein, we aimed to identify functional variants of GRK5 using patients diagnosed with a situs anomaly with CHD.

Several genetic analyses, including GWAS studies, have investigated an impact of *GRK5* variations on the cardiovascular system^5^,^6^,^7^,^8^,^9^. Those studies have concentrated on adult individuals and omitted patients with CHD. Here, we fill this gap and provide data on additional GRK5 variants which have not been previously described. Since we were interested only in the coding region of GRK5, we performed Sanger sequencing of exons. Using this approach we found 8 rare missense variants plus the previously reported p.Q41L allele^5^. We chose to further investigate three rare and highly conserved variants with a low SIFT score and a Polyphen-2 prediction indicating an impairment in function. In addition, we included the known p.Q41L variant as it has previously been shown to be more potent in receptor desensitisation than wild-type GRK5^5^,^24^.

To evaluate the functionality of these variants, and therefore the possibility they are risk alleles, we tested four characteristics of GRK5: the ability to desensitise GPCRs, predominant localisation to the plasma membrane or the cilium and finally whether the variants would be able to rescue the lateralisation defect upon Grk5l knockdown in zebrafish embryos. Consistent with previous data we found that p.Q41L was functional, if not even more potent, in terminating G protein-mediated signalling of GPCRs^24^. This could be explained by the position of the variant, which is in the membrane interface (Fig. 1b). Since leucine likely binds better to the membrane than glutamine, there may be more GRK5 readily available for phosphorylation of activated receptors. Interestingly, however, the p.Q41L variant failed to rescue Grk5l knockdown embryos, despite robust expression in zebrafish. This controversy mirrors other discrepancies regarding p.Q41L, which appears to prevent negative outcomes in certain heart failure patients^5^ while predisposing at the same time to other cardiovascular conditions such as atrial fibrillation^7^,^9^. It also suggests that GRK5 variants may confer different outcomes depending on the cell type, disease state, interaction partner or the signalling cascade. Further studies will be needed to better understand those context-dependent actions of p.Q41L.

What about the other three rare variants? Recently, the crystal structure of GRK5 has been published^26^,^27^. According to that, p.G298S is likely to destabilize the kinase domain as the side chain of the serine would introduce steric clashes. Consistent with that p.G298S acted as a non-functional mutant in all assays tested. p.R304C and p.S425M, however, reside in areas of GRK5 that are conserved among GRKs and are solvent exposed. Thus their potential roles in folding or function are unknown. In light of this, some of the results we obtained are unexpected. Both, p.R304C and p.S425M retained the canonical GRK ability to desensitise receptors. During left-right asymmetry development however, which is disturbed in heterotaxy patients, p.R304C appears to be non-functional. Thus, depending on the conformational flexibility of GRK5 any of the three variants may reside in a crucial domain. Interestingly, we also observed that p.R304C cannot be expressed to high levels in cells, which explains the reduced activity in receptor desensitization. In contrast to this, it is nicely expressed in zebrafish upon RNA injection. This suggests an additional regulation at the transcriptional or post-transcriptional level in the presence of this SNP. Having said that, even when using capped RNA, we did not obtain a rescue of asymmetry development by p.R304C implying some functional deficiency in addition to an impairment of plasmid-driven expression. These data are somewhat in accordance with the prediction of Polyphen-2, which classified this mutation as probably damaging.

p.S425M, on the other hand, could be expressed at high levels in cells and fish. It functioned very similar to wild-type GRK5 in the cellular assays. When co-injected with Grk5l MO it resulted in a rescue in left-right asymmetry development with respect to abdominal organ placement and heart looping. This is in stark contrast to the prediction of PolyPhen-2, which classified this mutation as possibly damaging.

The most intriguing variant is p.G298S. This alteration lies within the kinase domain of GRK5, but not in close proximity to the ATP pocket^26^,^27^. Nevertheless, p.G298S was classified as probably damaging by Polyphen-2 and had the lowest score using the SIFT algorithm, which suggested that it would show the most pronounced loss of function in our experiments. This was indeed the case: exchange of glycine for serine at position 298 renders cytoplasmic distribution of the kinase, although the domains relevant for association with the membrane’s phospholipids are in the outer most termini of GRK5^25^,^26^,^27^. One explanation for this could be that p.G298S is not folded correctly and thus retained in the cytoplasm. Secondly, p.G298S is unable to inhibit GPCR signalling, suggesting that unlike GRK2 it remains in the cytosol even upon receptor activation. Last, but not least, p.G298S is incapable of correcting aberrant *spaw* expression in Grk5l knockdown embryos and unable to rescue normally lateralized organogenesis. Together, these data suggest that p.G298S is a loss-of-function variant of GRK5 and potentially confers moderate-risk to the development of CHD.

CHDs associated with heterotaxy have only been survivable with the advances of surgical approaches over the last several decades. From an evolutionary genetic standpoint, this indicates that genetic causes of heterotaxy would be biased toward de novo mutations in affected individuals or combinatorial interactions of rare susceptibility alleles. Heterozygous rare variants identified in GRK5 are good candidates to support the latter mechanism. We have investigated deletions as well as gene duplications in heterotaxy^36^ and reviewed the data relating to CHD in general^37^. The majority of genetic causes identified so far show reduced penetrance and variable expressivity, which implies high genetic complexity^38^. It is very likely that these specific types of CHD result in some cases from the inheritance of multiple susceptibility alleles that disrupt molecular signalling in a synergistic fashion which in turn can be further worsened by additional environmental factors such as exposure to teratogens during pregnancy^39^. CHDs are typically thought to be inherited as a complex trait, and Mendelian inheritance patterns are rare. Thus, identifying potential susceptibility alleles for CHDs is an important goal for delineation of genetic contribution and risk assessment^40^. Furthermore, variants which are rare in the population are often predicted to be potentially damaging by bioinformatics prediction programs. The results presented on several genetic variants of *GRK5*, a gene previously identified as important for left-right patterning and normal heart development, indicate the complexity of dissecting allelic function *in vitro* and *in vivo*. Based on our data in zebrafish heart looping experiments we speculate that the p.G298S variant in GRK5 confers moderate susceptibility to CHD and situs abnormalities and may propagate the disease in combination and interaction with other susceptibility alleles and environmental factors. Overall, our results show that multiple functional assays may be required to determine allelic effects not only GRK5, but most likely also of other candidate genes and indicate zebrafish as a robust model for dissecting the complex human variations and combinatorial interactions underlying protein function and potentially heterotaxy.

## Methods

### Materials

Cell culture media and supplements were purchased from Life Technologies Invitrogen and Sigma. JetPrime^®^ was from Polyplus. CXCL12 and CCL11 were purchased from Peprotech.

### Patient Cohorts and Sequencing

Two heterotaxy cohorts with concomitant CHD were analysed: DNA samples of the first cohort (n = 69) were provided by the National Register for Congenital Heart Defects (Berlin, Germany) (collection and distribution were approved by the local ethics committee in Berlin). Here, according to German laws no ethnic data was collected. Medical records regarding the heart phenotype and situs were provided with the samples. These samples were screened for potential genetic variations in all 16 exons of GRK5 as well as for known mutations in genes associated with situs defects, namely *ZIC3, ACVR2B, LEFTY2, CFC1, NODAL* and *FOXH1*. A second cohort (n = 118) with phenotype information including imaging and diagnostic studies, clinical genetic testing results, and pathology results were collected at Cincinnati Children’s Hospital Medical Center (CCHMC) under a protocol approved by the CCHMC Institutional Review Board. In these probands, exons 2, 5, 9, 13, 14 and 15 of *GRK5* were screened. This study was approved by the local ethics committee at Ulm University and by the CCHMC Institutional Review Board. The study was performed in accordance with the Declaration of Helsinki protocols. Prior to inclusion into the study patients or their respective parents gave their informed consent.

DNA samples from whole blood were isolated by standard procedures. For gene analysis, we designed intronic primers to PCR amplify (ReadyMix Taq PCR Mix, Sigma Aldrich or FastStart PCR Master, Roche Applied Sciences) all coding exons and the exon-intron boundaries of *GRK5, ZIC3, ACVR2B, LEFTY2, CFC1, NODAL* and *FOXH1*. Primer pairs for the amplification of coding exons, and the approximately 50 base pairs (bp) of flanking UTR or intronic sequences, are available upon request. The purified PCR products were sequenced using BigDye terminator version 3.1 chemistry on an ABI3700 Genetic Analyzer (Applied Biosystems). Sequence traces were assembled, aligned, and analysed using Sequencher (Gene Codes) or Mutation Surveyer (SoftGenetics) software.

### Cloning

For experiments in zebrafish N-terminally HA-tagged Grk5l was cloned into pCS2+ and pCS2+ GFP via EcoRI. Site-directed mutagenesis was performed with a PCR based approach and capped RNA was prepared using Ambion’s mMessage mMachine Kit from linearised plasmids. For subcellular localization studies in HEK293 cells and hTert immortalised fibroblasts N-terminally tagged wild-type and variant Grk5l was cloned into pEGFP-N3 via BamHI and HindIII. cDNAs of human CXCR4 (accession number NM_001008540) and human CCR3 (accession number XM_006712960) were amplified from spleen and monocyte cDNA and cloned into pcDNA3.1 + with a N-terminal HA-epitope. To generate chimeric Gα_qi5_ in pcDNA3.1+, the last five amino acids of Gα_q_ in EE-epitope-tagged human Gα_q_ (GNA0Q0EI00, UMR cDNA Resource Center) were exchanged for the sequence encoding Gα_i2_ (according to Conklin *et al*.^41^). The reporter plasmids pSRE.L and pRL-TK were obtained from Dr. Dianqing Wu (New Haven, CT) and Promega, respectively.

### Cell Culture

HEK293 cells were maintained at 37 °C in a humidified atmosphere containing 5% CO_2_ in Dulbecco´s modified Eagle´s medium (DMEM, Sigma Aldrich) supplemented with 10% (v/v) fetal calf serum (FCS), 100 units/ml penicillin, 100 μg/ml streptomycin, 2 mM L-glutamine, 25 mM HEPES, and 1 mM sodium pyruvate. MEMα (Life technologies) supplemented with 10% FCS, 100 units/ml penicillin and 100 μg/ml streptomycin was used to culture hTert immortalised fibroblasts. Cilia formation in fibroblasts was induced by a 48 hours culture in MEMα containing 0.1% FCS.

### Desensitization Assay

Luciferase assays were performed using the Dual-Luciferase^®^ Reporter Assay System (Promega) as described previously^42^. HEK293 cells were seeded into 24- or 48-well plates at a density of 1,6 × 10^5^ and 8 × 10^4^ cells per well, respectively, and were grown for 24 h in 1 ml or 0.5 ml of the same medium per well prior to transfection. For transfection with JetPrime^®^ (Polyplus-transfection Inc.), plasmid DNA was mixed with 2 μl transfection reagent per μg of DNA in transfection buffer. In each transfection, the amounts of DNA were kept constant between the samples by adding the corresponding empty vector. Plasmid amounts transfected were: 30 ng per well of pSRE.L and pRL-TK, 300 ng per well of either empty vector pcDNA3.1 + and vector pcDNA3.1 + encoding Gαqi5 (Control-Mock, 50 ng/well), 200 ng per well murine AT1a^43^, 200 ng per well HA-epitope-tagged CCR2b, 200 ng per well HA-epitope-tagged pCXCR4, 200 ng per well HA-epitope-tagged pCCR3, and 100 ng per well of the individual GRK5 plasmids. The plasmid encoding mouse At1a was a kind gift of Lutz Hein. Cells were transfected with the reporter gene plasmid pSRE.L that carries the firefly luciferase gene under the control of a modified transcriptional regulatory element, referred to as SRE.L. To correct for variations in transfection efficiency, cells were co-transfected with a reporter plasmid pRL-TK, carrying a *Renilla reniformis* luciferase gene under the control of the Herpes simplex virus thymidine kinase (HSV-TK) promoter, which provides low-level, constitutive expression of the *Renilla* luciferase. Twenty-four hours after transfection, cells were stimulated with the indicated chemokine ligands and incubated for another 7 hours. After a single wash with 0.5 ml buffer A (10 mM Na_2_HPO_4_, 1.8 mM KH_2_PO_4_, 140 mM NaCl, 2.7 mM KCl, pH 7.4), the cells were lysed with 80 μl (24-well plates) or 40 μl (48-well plates) Passive Lysis Buffer (Promega) under gentle agitation on a rocking plate for 15 min at room temperature. The lysate was centrifuged for 10 min at 12,000 × g and the supernatants were collected for luciferase assays. For measurement of reporter gene activity, 10 μl of cleared lysate was combined with 25 μl of Luciferase Assay Reagent II to record the firefly luciferase activity. Twenty-five μl of Stop & Glo^®^ Reagent was added to allow determination of *Renilla* luciferase activity.

Data from representative experiments are shown as means ± standard deviation of triplicate determinations performed on independently transfected cells.

### Western Blotting

HEK293 cells transfected as for the signalling assay were lysed in a SDS based lysis buffer^44^ and treated with Benzonase (Pierce Thermo Fisher). Proteins were separated on Bolt^®^ 4–12% Bis-Tris Plus Gels (Life technologies) and blotted onto nitrocellulose. Primary antibodies to detect GRK5 (ARP54750_P050, 1:1000) and GAPDH (clone 6C5, 1:500) were purchased from Aviva Antibodies and Acris antibodies, respectively. Signals were obtained using near infrared-labelled secondary antibodies (Li-COR) and a Li-COR Odyssey SA system.

### Subcellular localization studies

HEK293 cells and fibroblasts were transfected with Attractene Transfection reagent (Qiagen) according to the manufacturer’s instructions (0.5 μg plasmid per well of a 6-well plate). Fertilized eggs were injected using capped RNA encoding GFP fusion constructs of wild-type Grk5l and Grk5l variants (see also section below). Cilia in fibroblasts were immunostained using a mouse-anti-acetylated antibody (1:500, clone 6-11B-1, Sigma) and an Alexa568-labelled secondary antibody. Cells as well as injected embryos were mounted in Vectashield mounting medium (Vectorlabs).

### Zebrafish Husbandry, Manipulation and Analysis

All zebrafish procedures in this study followed the protocols approved by local authorities in Germany (registry no. 0140) and complied with the guidelines from Directive 2010/63/EU of the European Parliament on the protection of animals used for scientific purposes. Zebrafish were maintained in a water-recycling tank system (Tecniplast) and exposed to a 14 hours light and 10 hours dark cycle. Embryos generated from natural wild-type matings (EK and AB strains) were injected at the 1–2 cell stage using an Eppendorf Femtojet and pulled glass capillaries. For both, rescue experiments as well as subcellular localization studies, 500 ng capped RNA was injected into the yolk. Rescue RNAs encoded HA-tagged Grk5l or variants thereof, that was *in vitro* transcribed from pCS2+ plasmids. GFP-tagged RNAs were transcribed from pCS2+ GFP vectors (a vector map is available on request). The MO to knock down Grk5l has been extensively validated and described previously^10^,^45^. After injection embryos were allowed to develop at 28.5 °C to the desired stages, before they were fixed for subsequent analysis. Whole mount *in situ* hybridisation was performed according to standard protocols using a DIG-labelled RNA probe against *southpaw* (*spaw*)^10^,^46^.

### Microscopy

Transfected cells were analysed with a Leica TCS SP5II confocal microscopy. Single plane pictures were taken. Zebrafish embryos were either imaged using a Leica MZ 125 equipped with an IC80 HD or by confocal microscopy.

### Statistical Analysis

Due to the restriction that no ethnic data may be recorded in Germany, no statistical analysis was done for the prevalence of *GRK5* variants. Statistical analysis for functional assessment of the variants was performed in Prism4 or Prism6 (GraphPad Software Inc.). The precise statistical test as well as number of experiments and/or animals is indicated in the respective figure legends. Bar graphs are presented as means ± SEM.

## Additional Information

**How to cite this article**: Lessel, D. *et al*. The analysis of heterotaxy patients reveals new loss-of-function variants of *GRK5*. *Sci. Rep*. **6**, 33231; doi: 10.1038/srep33231 (2016).

## Supplementary Material

Supplementary Information

## Figures and Tables

**Table 1:** *GRK5* variants in patients with CHD and concomitant situs defect.

Patient no.	Cohort	Sex	CHD	Situs	GRK5 variant
1	GER	M	DILV	SI	rs55980792
8	GER	F	ccTGA	SI	rs2230349
9	GER	F	AVSD (imb.)	RI	rs17098707
19	GER	F	DORV-TGA Typ	DEX	rs2230349
21	GER	F	PA + VSD	DEX	rs55980792
22	GER	F	PAPVC	DEX	rs17098707
27	GER	F	AVSD	DEX	rs2230349
38	GER	F	AVSD (imb.)	RI	rs2230349, G549R/wt
39	GER	F	AVSD	DEX	rs149159651
41	GER	M	VSD	DEX	P464S/wt
43	GER	F	ccTGA	SI	rs55980792, rs140946236
45	GER	M	AVSD (imb.)	SI	rs2230349
67	GER	M	DORV-TGA-Typ	DEX	rs2230349
1	Cin	F	Unknown CHD	HTX	rs17098707 homozygous
2	Cin	M	TA, ASD	HTX	rs17098707
3	Cin	M	AVSD (imb)	RI	rs17098707
4	Cin	F	TGA, TAPVC	DEX	rs17098707
5	Cin	M	AVSD (imb), PA, PAPVC	RI	rs17098707; rs145397190
6	Cin	F	None	DEX	rs17098707
7	Cin	F	unknown CHD	HTX	rs17098707
8	Cin	F	PAPVC, IVC	HTX	rs17098707
9	Cin	M	UVH	RI	rs77323445
10	Cin	M	IVC	LI	rs77323445

CHDs and situs defects as diagnosed in the German (GER) or Cincinnati (Cin) cohort for those individuals tested positive for a *GRK5* variant. All variants were heterozygous, if not indicated otherwise. M, male; F, female, DEX, dextrocardia; HTX, heterotaxy; LI, left isomerism (polysplenia); RI, right atrial isomerism (asplenia); SI, situs inversus; imb., imbalanced. For explanations of the precise CHD we refer to Supplementary Table S1. A complete list of all 69 German patients can be found in Supplementary Table S2.

**Table 2:** Description and incidence of *GRK5* variants both cohorts.

Variant	Variant ID	Exon	SIFT score	Polyphen-2	German patients (n = 69)	Cincinnati patients (n = 118)	1000 Genomes	EVS	ExAc information		No. of homozygotes	Allele frequency (%)
					MAF (%)	MAF (%)	MAF (%)	MAF (%)	Allele count	Allele no.		
p.Q41L	rs2230345	2	0.15	benign	1.45	4.66	8.80*	9.71*	5214	120928	434	4.312
p.A119V	rs55980792	5	0.03	benign	2.17	0.00	0.30	0.93	845	121374	2	0.6962
p.G298S	rs140946236	9	0.00	probably damaging	0.72	0.00	0.00	0.02	21	118708	0	0.01769
p.R304C	rs145397190	9	0.01	probably damaging	0.00	0.42	0.00	0.14	56	116430	1	0.0481
p.R304H	rs2230349	9	0.03	benign	4.34	9.32	11.90	7.37	12870	116318	882	11.06
p.T425M	rs77323445	13	0.04	possibly damaging	0.00	0.85	1.20	1.14	391	116810	8	0.3347
p.P464S	not annot.	13	—	benign	0.72	0.00	—	—	—	—	—	—
p.D495D	rs149159651	14	n/a	n/a	0.72	0.00	0.00	0.12	133	120158	0	0.1107
p.G549R	not annot.	15	—	probably damaging	0.72	0.00	—	—	—	—	—	—

^*^MAF for p.Q41L below 2% in probands of European descent.

Table listing detected genetic variants of GRK5, the SIFT scores and the Polyphen-2 predictions regarding the impact of the mutation on GRK5’s function, the minor allele frequencies (MAF) across all ethnicities of all detected genetic variants of GRK5 in the 1000 Genomes, in the Exome Variant Server (EVS) and in the Exome Aggregation Consortium (ExAC). At the time of submission, p.P464S and p.G549R have not been annotated, yet, by large scale sequencing efforts.
