# Supplementary material for: The analysis of heterotaxy patients reveals new loss-of-function variants of GRK5
Source: Sci Rep. 2016 Sep 13;6:33231. doi: 10.1038/srep33231 (PMC5020398; doi:10.1038/srep33231)
Supplement: Supplementary Information [file srep33231-s1.pdf]

The analysis of heterotaxy patients reveals new loss-of-function variants of *GRK5*

Davor Lessel[1#], Tariq Muhammad[2#¶], Teresa Casar Tena[3], Barbara Moepps[4], Martin D Burkhalter[3], Marc-Phillip Hitz[5], Okan Toka[6], Axel Rentzsch[7], Stephan Schubert[8], Adelheid Schalinski[9], Ulrike MM Bauer[9], Christian Kubisch[1], Stephanie M Ware[2] and Melanie Philipp*[3]

Supplementary Table S1: CHDs as diagnosed

Abbreviation	Full name of heart defect
ASD	Atrial septal defect
AVSD	Atrioventricular septal defect
DILF	Double inlet left ventricle
DIRV	Double inlet right ventricle
DORV	Double outlet right ventricle
IVC	Inferior vena cava abnormality
LSVC	Persistent left superior vena cava
PA	Pulmonary atresia
PAPVC	Partial anomalous pulmonary venous connection
PDA	Persistent ductus arteriosus Botalli
PS	Pulmonary stenosis
TA	Tricuspid atresia
TAC	Truncus arteriosus communis
TAPVC	Total Anomalous Pulmonary Venous Connection
ccTGA	congenitally corrected TGA
TGA	Transposition of great arteries
TOF	Tetralogy of Fallot
VSD	Ventricular septal defect
UVH	Univentricular heart

List of abbreviations of congenital heart defects. Patients' side diagnoses were omitted from the lists.

Supplementary Table S2: Complete list of German cohort

Patient no.	Sex	CHD	Situs	GRK5 variant
1	M	DILV	SI	rs55980792 (het)
2	F	DORV-TGA Typ	RI	rs2275036 (het)
3	M	DILV	DEX	
4	F	AVSD (imb.)	DEX	rs2275036 (het)
5	F	DILV	DEX	
6	M	DORV-TGA Typ	DEX	rs2275036 (het)
7	M	DILV	SI	
8	F	ccTGA	SI	rs2230349 (het)
9	F	AVSD (imb.)	RI	rs17098707 (het)
10	F	DIRV	LI	rs2275036 (het)
11	F	AVSD (imb.)	SI	
12	F	AVSD (imb.)	RI	
13	M	ccTGA	DEX	
14	M	ccTGA	DEX	
15	F	DORV	DEX	
16	M	TGA	SI	rs2275036 (het)
17	F	PDA	HTX	
18	F	DILV	SI	
19	F	DORV-TGA Typ	DEX	rs2230349 (het)
20	F	ccTGA	SI	
21	F	PA+VSD	DEX	rs55980792 (het)
22	F	PAPVC	DEX	rs17098707 (het)
23	M	ccTGA	SI	
24	F	ccTGA	SI	rs2275036 (het)
25	M	TOF	DEX	rs2275036 (het)
26	M	ccTGA	RI	
27	F	AVSD	DEX	rs2230349 (het)
28	M	VSD	DEX	rs2275036 (het)
29	F	AVSD (imb.)	RI	
30	F	ccTGA	DEX	
31	M	ccTGA	DEX	rs2275036 (het)
32	F	ccTGA	DEX	
33	M	AVSD (imb.)	RI	
34	M	AVSD+TOF	SI	
35	F	ccTGA	DEX	
36	M	PAPVC	DEX	
37	M	ccTGA	DEX	rs2275036 (het)

38	F	AVSD (imb.)	RI	rs2230349 (het), G549R/wt
39	F	AVSD	DEX	rs149159651 (het)
40	M	AVSD (imb.)	SI	
41	M	VSD	DEX	P464S/wt
42	M	ccTGA	DEX	
43	F	ccTGA	SI	rs55980792 (het), rs140946236 (het)
44	F	AVSD (imb.)	LI	
45	M	AVSD (imb.)	SI	rs2275036 (het), rs2230349 (het)
46	F	DORV-TGA Typ	DEX	
47	F	AVSD (imb.)	RI	rs2275036 (het)
48	F	PS	HTX	
49	F	TAC	SI	
50	F	ccTGA	SI	
51	F	ccTGA	SI	rs2275036 (het)
52	F	PAPVC	DEX	
53	F	PA + VSD	DEX	
54	F	UVH	DEX	rs2275036 (het)
55	F	UVH	RI, DEX	
56	M	PAPVC	DEX	
57	M	UVH	DEX	
58	F	PAPVC	DEX	
59	F	ccTGA	SI	
60	M	LSVC	DEX	
61	M	PAPVC	DEX	
62	M	PAPVC	DEX	
63	M	ccTGA	DEX	
64	M	AVSD (imb.)	DEX	
65	F	UVH	DEX	
66	M	UVH	SI	
67	M	DORV-TGA-Typ	DEX	rs2230349 (het)
68	F	VSD	DEX	
69	F	PAPVC	DEX	rs2275036 (het)

Complete list of CHD, situs diagnosis and detected *GRK5* variants in the German cohort. Imb., imbalanced; DEX, dextrocardia; LI, left isomerism (left-sided organs are dominant in this condition at the expense of right sided organs; is apparent by multiple spleens that are smaller than normal); SI, situs inversus; RI, right isomerism (a condition, where the patients has “two right sides”, but no spleen).

Supplementary Figure S1: Amino acid alignment of Grk5l and human GRK5.

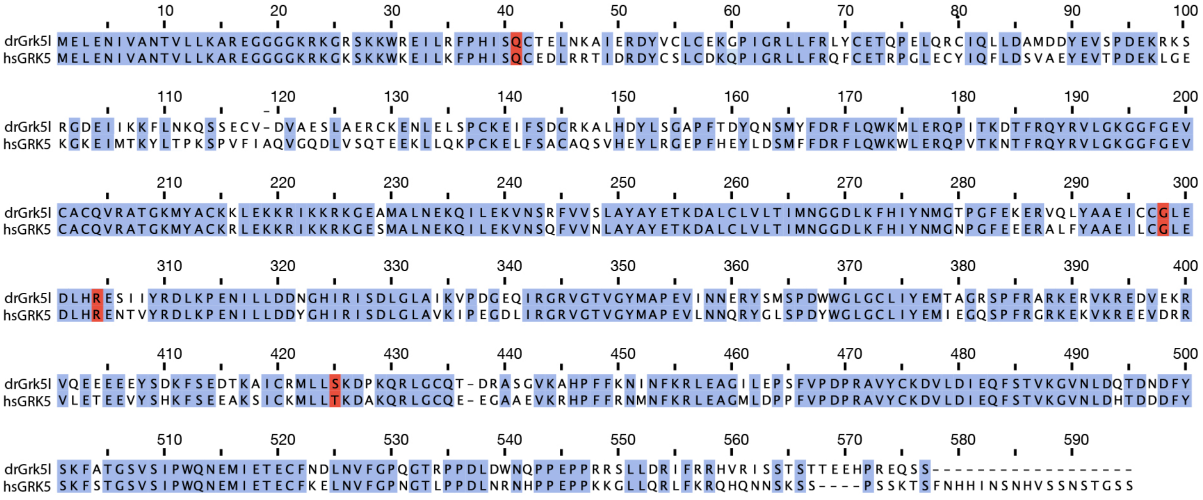

Alignment of zebrafish Grk5l and human GRK5. Identical amino acids are labelled in blue. In red those residues are highlighted which were mutated in patients and analysed in this study. The alignment was done with the help of http://multalin.toulouse.inra.fr/multalin/.

Supplementary Figure S2: ***Southpaw*** **expression upon injection of Grk5l variants.**

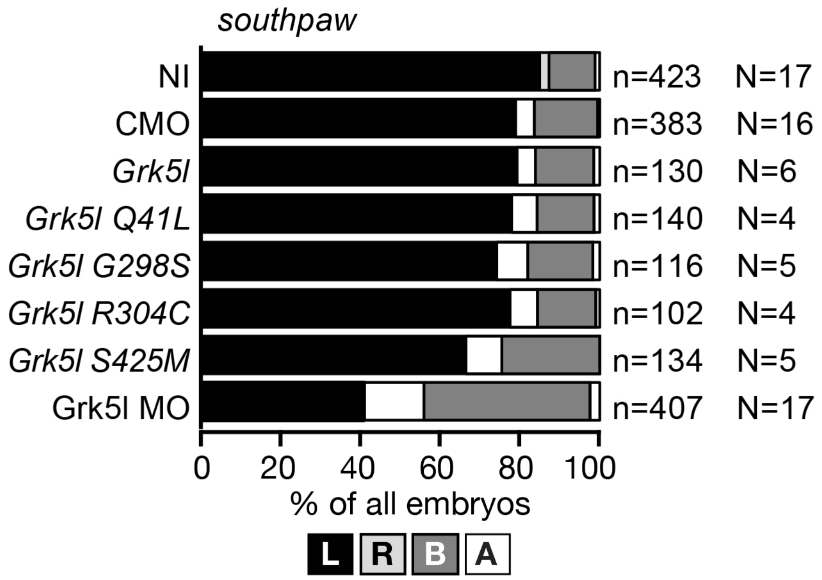

Analysis of *southpaw* expression in the lateral plate mesoderm (LPM) of zebrafish embryos upon injection of capped RNAs encoding wild-type Grk5l (*Grk5l*) or different variants thereof. For comparison, the same controls (NI, non-injected; CMO, control MO injected; Grk5l MO, embryos injected with a knockdown MO against Grk5l) as in the main Figure 5 are shown. n indicates the number of individual embryos per condition and N the number of independent experiments. L, expression in the left LPM; R, expression in right LPM; B, expression on both sides of the midline; A, no expression.

Supplementary Figure S3: Pancreas position upon injection of Grk5l variants.

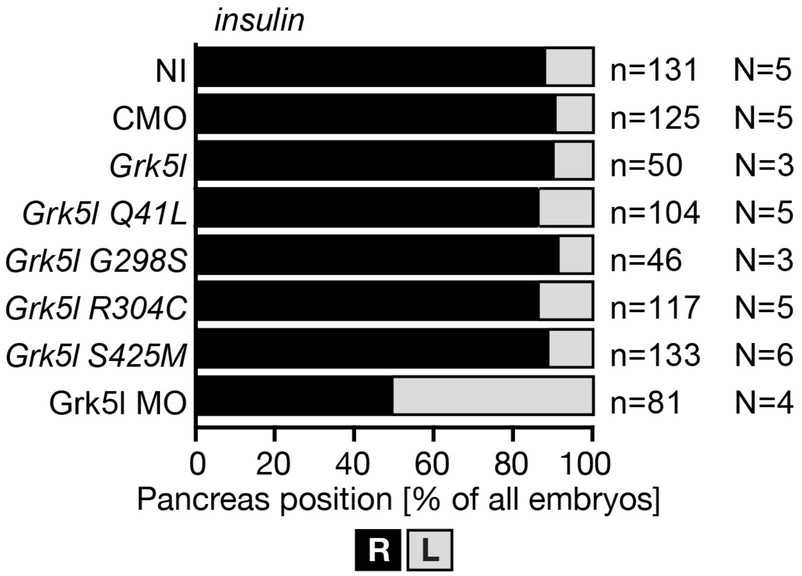

Analysis of *insulin* expression to determine pancreas position of zebrafish embryos upon injection of capped RNAs encoding wild-type Grk5l (*Grk5l*) or different variants thereof. For comparison, the same controls (NI, non-injected; CMO, control MO injected; Grk5l MO, embryos injected with a knockdown MO against Grk5l) as in the main Figure 5 are shown. n indicates the number of individual embryos per condition and N the number of independent experiments. R, pancreas localized right from midline; L, pancreas localized left from midline.

Supplementary Figure S4: Heart looping upon injection of Grk5l variants.

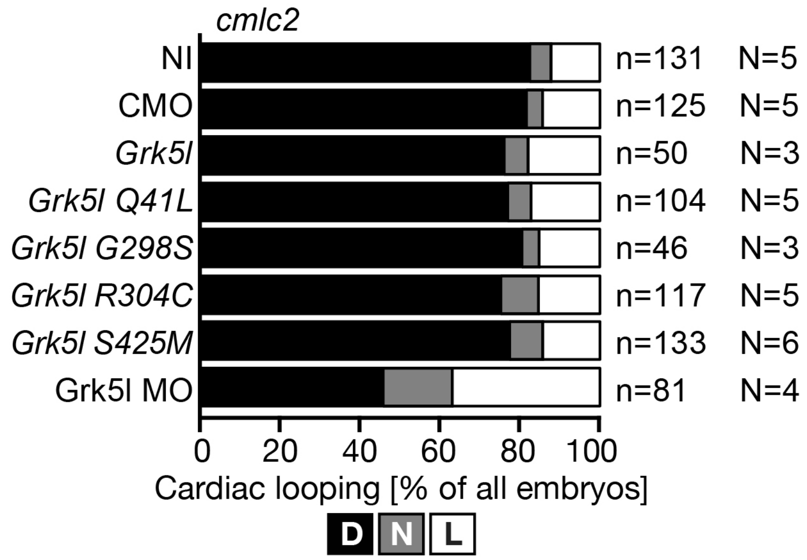

Analysis of *cardiac myosin light chain 2* (*cmlc2*) expression to determine heart looping in zebrafish embryos injected with capped RNAs encoding wild-type Grk5l (*Grk5l*) or different variants thereof. For comparison, the same controls (NI, non-injected; CMO, control MO injected; Grk5l MO, embryos injected with a knockdown MO against Grk5l) as in the main Figure 5 are shown. n indicates the number of individual embryos per condition and N the number of independent experiments. D, correct D-loop; N, unlooped heart; L, inversely looped heart (N-loop).
